# Effects of Lactic Acid Bacteria Fermentation and In Vitro Simulated Digestion on the Bioactivities of Purple Sweet Potato Juice

**DOI:** 10.3390/foods13244094

**Published:** 2024-12-18

**Authors:** Yingjia Tong, Zeqing Wang, Qunyi Tong, Yutong Liu

**Affiliations:** 1 School of Life Sciences and Health Engineering, Jiangnan University, 1800 Lihu Avenue, Wuxi 214122, China; 2 State Key Laboratory of Food Science and Technology, Jiangnan University, Wuxi 214122, China; 6210112160@stu.jiangnan.edu.cn (Z.W.); tqyjn@163.com (Q.T.); yutongliu2024@126.com (Y.L.); 3 Synergetic Innovation Center, Jiangnan University, Wuxi 214122, China; 4 School of Food Science and Technology, Jiangnan University, Wuxi 214122, China

**Keywords:** purple sweet potato juice, in vitro simulated digestion, phenols bioactivities, bioaccessibility and bioavailability, antioxidant activity, lactic acid bacteria

## Abstract

The effects of lactic acid bacteria fermentation and in vitro simulated digestion on phenolic bioavailability, phenolic bioavailability, and antioxidant activity of purple sweet potato juice (PSPJ) were investigated. The PSPJ was fermented by *Lactobacillus rhamnosus* and *Streptococcus thermophilus*. The viable bacterial count, phenolic components, antioxidant activity, phenolic bioaccessibility, and phenolic bioavailability of PSPJ were analyzed during the simulated digestion process in vitro. The data displayed that lactic acid bacteria fermentation increased total α-glucosidase inhibition, total flavonoid content, and ratephenolic content. The antioxidant activities were improved after in vitro simulated digestion due to the biotransformation of phenolic substances by lactic acid bacteria fermentation. The bioaccessibility and bioavailability of phenols in PSPJ were improved with fermentation of lactic acid bacteria. Furthermore, the viable bacteria count of the two strains was significantly improved (>7 log CFU/mL) after simulated digestion in vitro.

## 1. Introduction

Purple sweet potato (*Ipomoea batatas* L.) is an herbaceous plant of the genus *Ipomoea* in the family *Cyclophyllaceae* [1]. Purple sweet potatoes are rich in nutrients, including carbohydrates, dietary fibers, vitamins, and minerals, but also have bioactive compounds such as anthocyanins, phenolic acids, and organic acids [2]. It has been demonstrated that these bioactive compounds have a variety of physiological functions, such as antioxidant, cardiovascular disease prevention, anti-inflammatory, and memory-enhancing effects [3], so purple sweet potatoes have a broad development prospect. Polyphenols are often found in the bound state (combined with protein, dietary fiber, and so on) in food, making it difficult to be absorbed and utilized [4]. When purple sweet potatoes are consumed and digested by the human body, only a small amount of the polyphenols reach the colon for absorption, and their phenolic bioaccessibility and phenolic bioavailability are poor [5]. Therefore, their processing method needs to be further researched.

One common method for fabricating functional foods is fermentation by lactic acid bacteria (LAB). Non-dairy drinks based on fruits and vegetables have attracted a lot of attention because they are nutritious, pleasant to taste, and attractive options, especially for groups allergic to dairy drinks [6]. LAB fermentation can improve the nutritional value and functional properties of fruit and vegetable juice. The various enzymes produced by lactic acid bacteria fermentation can break down complex phenols into small molecules that are easier to digest and absorb, thereby improving the phenols’ bioavailability [7]. In addition, LAB fermentation (*Streptococcus lactis* and *Pediococcus pentosaceu*) can enhance the stability of phenols due to the decrease in pH during fermentation, thereby improving the phenols bioaccessibility of blueberry puree during in vitro digestion process [4]. The carbohydrate-rich nature of PSPJ makes it a good substrate for LAB growth. Briefly, LAB fermentation can be used to improve PSPJ’s antioxidant activity, phenols bioaccessibility, and phenols bioavailability. In vitro simulated digestion models are often used to study changes in food composition during the digestion process [8]. However, few studies have examined the effects of LAB fermentation on the phenolic composition, phenols bioaccessibility, antioxidant activities, and phenols bioavailability of PSPJ during the in vitro simulated digestion process. Therefore, this is a new attempt to explore the changes in the bioactivity of purple sweet potato by using in vitro digestion model, which is helpful in understanding the health benefits of the bioactive ingredients in purple sweet potato.

In summary, two strains of *Lactobacillus rhamnosus* and *Streptococcus thermophilus* were chosen for fermentation to enhance the antioxidant activity, phenolic bioaccessibility, and phenolic bioavailability of PSPJ. The variations in viable bacteria count, phenolic content, and antioxidant activity of PSPJ during in vitro simulated digestion were studied. In addition, the inhibitory activity of α-glucosidase during fermentation of PSPJ was determined, and the bioaccessibility and bioavailability of phenol substance in PSPJ were studied. These studies provided strong support for the progress of LAB fermentation of PSPJ.

## 2. Materials and Methods

### 2.1. Materials

Purple sweet potatoes were purchased by the markets in Zhongxiang City (Jingmen, China). Medium temperature α-amylase (≥3000 U/mL), glucoamylase (≥10,000 U/mL), *Lactobacillus rhamnosus* GDMCC 1.1732 (Lr), and *Streptococcus thermophilus* GDMCC 1.1808 (St) were stored in our lab [9]. Pepsin (≥2500 U/mg) and pancreatin (≥100 U/mg) were purchased by Innokai Technology Co., Ltd. (Beijing, China). Bile salt was obtained by Sinopharm Chemical Reagent Co., Ltd. (Shanghai, China). Other experimental reagents are from Aladdin Biochemical Technology Co., Ltd. (Shanghai, China).

### 2.2. Fermentation of PSPJ

PSPJ was fermented as a method described previously with LAB (Lr or St, 5 × 10^7^ CFU/mL, respectively) at 37 °C for 48 h [9]. Unfermented PSPJ was used as a control. Before and after fermentation, samples were taken and centrifuged to obtain supernatants for analysis.

### 2.3. In Vitro Simulated Digestion

In vitro simulated digestion, including simulated gastric digestion (SGD) and simulated intestinal digestion (SID), was experimentally adjusted on the basis of reported literature [10]. For the SGD, 50 mL of fermented and unfermented PSPJ were prepared separately into conical flasks. 200 mL of PBS buffer at pH 3 and 0.2 g pepsin (2000 U/mL) were introduced into a conical flask, followed by 2 h of SGD in an incubator (37 °C, 120 rpm). Then, the conical flask is placed in ice for 10 min to prevent pepsin digestion. Before the onset of SID, the pH was increased to 7 by the introduction of 1 M NaHCO_3_, followed by the addition of 0.3 g 100 U/mL pancreatin and 10 mM bile salt, and incubated in the incubator for 2 h (37 °C, 60 rpm. A dialysis bag was immersed in a beaker containing distilled water, and then NaHCO_3_ (1 M) was added. 20 mL of the intestinal digestive fluid was taken and placed in the beaker for 2 h (37 °C, 60 rpm). The solution in the dialysis bag was collected as an intestinal absorption solution and sampled after SGD and SID, respectively. Then the supernatant was centrifuged and stored at −20 °C for analysis.

### 2.4. Viable Counts of LAB

A typical strategy was adopted to estimate the number of viable bacteria in the LAB [11]. The detailed process was to inoculum samples with different dilution concentrations (0.1 mL) on the MRS agar plate, incubate at 37 °C for 48 h, and then collect the colony amount of a specific number of colonies.

### 2.5. Phytochemical Concentration Analysis

#### 2.5.1. Total Phenolic Content (TPC)

TPC was recorded according to the previous strategy with slight modifications [12]. Briefly, 1 mL of the diluted sample was introduced into a test tube containing 5 mL of distilled water, followed by 3 mL Na_2_CO_3_ solution and 2 times diluted 1 mL folin-phenol solution, and placed in the dark for 1 h, and absorbance was measured at 765 nm. Experimental data were presented as gallic acid equivalent (mg GAE/mL).

#### 2.5.2. Total Flavonoid Content (TFC)

TFC was evaluated using the previously reported strategy with minor modifications [11]. A 5% NaNO_2_ solution and a 10% Al(NO_3_)_3_ solution were introduced into the diluted sample (1 mL) and then stood for 6 min. Finally, 4 mL (1 M) of NaOH solution was added, diluted to 10 mL with a 60% ethanol solution, left in darkness for 15 min, and then displayed with rutin equivalent (mg RE/mL).

#### 2.5.3. Total Anthocyanin Content (TAC)

TAC was recorded using the pH-differential strategy [13]. Absorbance values (A) with 530~700 nm were recorded, and the TAC is calculated as follows:

$$A={\left(A_{530\;\mathrm{nm}}-A_{700\;\mathrm{nm}}\right)}_{\mathrm{pH}1.0}-{\left(A_{530\;\mathrm{nm}}-A_{700\;\mathrm{nm}}\right)}_{\mathrm{pH}4.5}$$

$$\mathrm{TAC}\;(\mathrm{mg}/L)=\left(A\times M_{w}\times \mathrm{DF}\times 1000\right)/\left(\varepsilon \times l\right)$$

Cyanidin-3-O-glucoside’s molecular weight (Mw) is 449.2 g/mol, its molar absorptivity (ε) is 26,900 L/(mol·cm), the path length (l) is 1 cm, and DF is the dilution factor.

### 2.6. Phenolic Profiles

The target data were obtained using the Waters ACQUITY BEH (100 × 2.1 mm, 1.7 μm) column described previously [14]. The mobile phase (A, B) consists of 0.1% (*v*/*v*) formic acid solution and 0.1% acetonitrile formic acid solution, respectively. The chromatographic gradients were as follows: 0–1 min, 95% A; 1–9 min, 95% to 5% (*v*/*v*) A; 9–14 min, 5% A; 14–14.1 min, 5–95% A; 14.1–20 min, 95% A. Flow rate, injection volume, and column temperature were set at 0.3 mL/min, 5 μL, and 25 °C. Chromatograms were monitored at 280, 360, and 520 nm wavelengths.

### 2.7. α-Glucosidase Inhibition Rate Analysis

The determination of α-glucosidase activity was slightly adjusted according to previous reports, specifically by adding 5 mL of samples into 0.1 mol/L phosphate buffer (PBS, pH6.9) for dilution and standing for 30 min [15]. In the sample group (A_3_), 100 μL of supernatant was added to the beaker, and then 50 μL of a-glucosidase solution (10 U/mL) was added and incubated at 37 °C for 15 min. 4-nitrophenyl-α-D-glucopyranoside (pNPG, 1 mmol/L, 50 μL) was added and incubated at 37 °C for 15 min. Finally, 1 mol/L Na_2_CO_3_ solution (100 μL) was introduced to terminate the reaction. In the sample blank group (A_4_), 50 μL PBS buffer was substituted for α-glucosidase solution. In the control blank group (A_2_), α-glucosidase was replaced by 50 μL PBS buffer, and supernatant was replaced by 100 μL PBS buffer. In the control group (A_1_), 100 μL PBS buffer was substituted for supernatant. Absorbances were measured at 400 nm. The specific calculation formula is as follows:

$$\;\mathrm{Inhibition}\;\mathrm{rate}\left(\%\right)=1-\left(A_{3}-A_{4}\right)/(A_{1}-A_{2})\times 100\%$$

### 2.8. Analysis of Antioxidant Activity

Specific test methods for target data are as follows [10]: DPPH solution (2 mL, 0.2 mM/L) was added into the diluted sample (2 mL), and the test tube was placed in the dark for 30 min. Distilled water was used as a control. The absorbances of distilled water (A_1_) and sample (A_2_) were measured at 517 nm, respectively. The specific calculation formula is as follows:

$$\mathrm{DPPH}\;\mathrm{radical}\;\mathrm{scavenging}\;\mathrm{rate}\;\left(\%\right)=\left(A_{1}-A_{2}\right)/A_{1}\times 100$$

The assessment of free radical scavenging activity of ABTS was slightly adjusted based on previous reports [12]. Firstly, ABTS (400.0 mg) and potassium persulfate (68.8 mg) were diluted to 100 mL and left for 12 h, and then PBS buffer was diluted to 734 nm with an absorbance of 0.70 ± 0.02, which was the ABTS working fluid. Take 0.4 mL sample and put it into a 10 mL test tube, then add 3.6 mL ABTS prepared, and place it in a dark place for 30 min. The absorbances of distilled water (A_1_) and sample (A_2_) were measured at 734 nm, respectively. The calculation results of ABTS free radical clearance were obtained by the following formula:

$$\mathrm{ABTS}\;\mathrm{radical}\;\mathrm{scavenging}\;\mathrm{rate}\;\left(\%\right)=\left(A_{1}-A_{2}\right)/A_{1}\times 100$$

### 2.9. Determination of Phenols Bioaccessibility and Phenols Bioavailability

Bioaccessibility was evaluated by the number of compounds available for intestinal absorption after in vitro simulated digestion, and bioavailability was evaluated by the degree of absorption into the human circulation. The formulas for calculating phenols bioaccessibility and phenols bioavailability were as follows [16]:

$$\mathrm{Phenolics}\;\mathrm{bioaccessibility}\;\left(\%\right)=C_{1}/C_{2}\times 100$$

$$\mathrm{Phenolics}\;\mathrm{bioavailability}\;\left(\%\right)=C_{3}/C_{1}\times 100$$

C_1_—The concentration of phenols retained after in vitro simulated digestion (mg/L)

C_2_—The concentration of phenols before in vitro simulated digestion (mg/L)

C_3_—The concentration of phenols in the dialysis bag (mg/L)

### 2.10. Statistical Analysis

Results from the study were shown as the mean with a standard deviation based on three independent trials. Variance analysis was executed using SPSS 26 software (IBM, Armonk, NY, USA), and *p* < 0.05 denoted a statistically significant difference.

## 3. Results

### 3.1. Variations in Viable Counts Throughout the In Vitro Simulated Digestion Process

**Figure 1:**
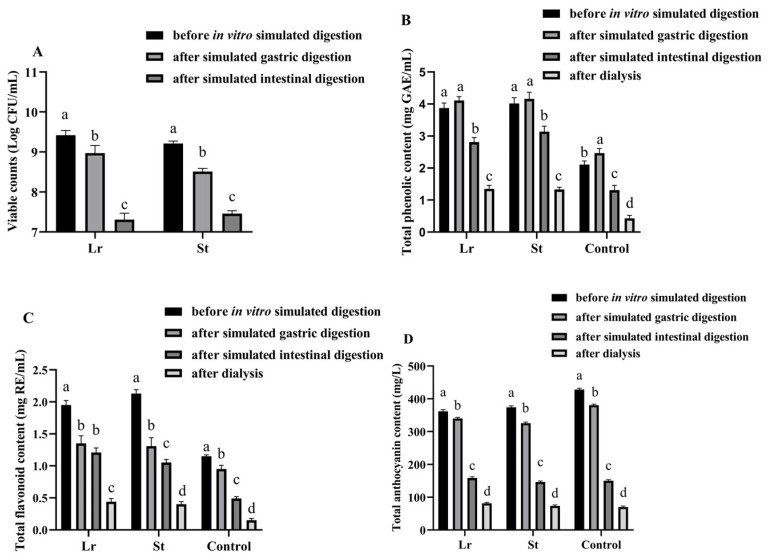
Changes in viable counts (**A**), TPC (**B**), TFC (**C**), and TAC (**D**) of PSPJ during the in vitro simulated digestion process. Values in the same pattern column with different superscript letters are significantly different (*p* < 0.05).

The viable count of LAB was an important indicator of beverages fermented by LAB [17]. After fermentation, the viable counts of Lr and St were 9.42 and 9.21 log CFU/mL, respectively, indicating that PSPJ was a suitable substrate for Lr and St (Figure 1).

The number of viable LAB was decreased during in vitro simulated digestion. This may be due to the lower pH in simulated gastric acid and the higher bile salt concentration in simulated intestinal fluid [18]. However, after in vitro simulated digestion, the viable counts of Lr and St were 7.31 and 7.46 log CFU/mL, respectively. This number reached a level that benefits health (7 log CFU/mL) [11].

### 3.2. Phytochemical Concentrations During In Vitro Simulated Digestion Process

It has been previously reported that antioxidant activity is positively correlated with the content of phenolic compounds [19]. After fermentation, there was a notable rise in TPC and TFC, as depicted in Figure 1. The St group showed higher TPC (4.02 mg GAE/mL) and TFC (2.13 mg RE/mL) than other groups. The increase in TPC and TFC may be due to the degradation of complex phenolic compounds by LAB fermentation [6]. Moreover, fermentation also led to the release of some bound phenols [20]. However, the TAC decreased from 428.13 mg/L to 361.56 and 374.32 mg/L after the fermentation of Lr and St, respectively, due to the enzymatic degradation of anthocyanins by LAB fermentation [21]. During the fermentation process, the activity of β-glucosidase could lead to the cleavage of anthocyanin glycosidic bonds. Therefore, some small phenolic acids were formed. Braga et al. utilized Lactobacillus delbrueckii to ferment the pulp of palm fruits, observing the conversion of cyanidin-3-glucoside and cyanidin-3-rutinoside into protocatechuic acid [22]. Similarly, Devi et al. employed the mixed fermentation of grape wine with lactic acid bacteria and yeast, resulting in the degradation of anthocyanins into malvidin, delphinidin, and peonidin, which were further degraded into syringic acid, gallic acid, or vanillic acid [23].

TPC was increased after SGD; however, it was decreased after SID. The St group showed the highest TPC (3.14 mg GAE/mL) among these groups after digestion. The increase in TPC during SGD might be caused by the enzymatic decomposition (pepsin) of the protein-polyphenol complex and the release of bound polyphenols [13]. However, the increase in pH during SID could make phenolic compounds unstable, which might undergo decomposition or structural changes, leading to a decrease in TPC [24]. After digestion, there was a significant decrease in TFC and TAC, with the Lr group having the highest values of TFC (1.21 mg RE/mL) and TAC (158.25 mg/mL). The reason might be that flavonoids were degraded by enzymes into phenolic acids and other compounds during the in vitro simulated digestion process [25]. After digestion, the fermented group exhibited higher TPC and TFC contents compared to the unfermented group.

### 3.3. Effects of In Vitro Simulated Digestion on Phenolic Composition

Research shows that polyphenols and antioxidant activity in food are significantly affected by simulated digestion in vitro [18]. After fermentation, the contents of caffeic acid, protocatechuic acid, and vanillic acid in the samples increased (Table 1). The Lr group had the highest caffeic acid content (24.24 mg/L), and the St group had the highest contents of vanillic acid (13.02 mg/L) and protocatechuic acid (5.08 mg/L). The increase in vanillic acid and protocatechuic acid content might be due to the enzymatic degradation (glycosidase) of peonidin-3-O-glucoside and anthocyanidin-3-O-glucoside by LAB to produce protocatechuic acid and vanillic acid [19]. However, the content of chlorogenic acids in the Lr and St groups decreased after fermentation, caused by the breakdown of chlorogenic acids into caffeic acid [26].

After in vitro simulated digestion, the contents of vanillic acid, syringic acid, protocatechuic acid, ferulic acid, and caffeic acid increased in the Lr group and St group. The St group showed the highest content of vanillic acid (15.13 mg/L), and the Lr group showed the highest content of ferulic acid (21.23 mg/L) and caffeic acid (37.14 mg/L) after digestion. During in vitro simulated digestion, free phenolic acids (like syringic acid) were released from protein polyphenol complexes under the action of proteases [12]. Acylated anthocyanins in PSPJ degraded during in vitro simulated digestion, producing free phenolic acids [27]. The increase in protocatechuic acid and vanillic acid might be caused by the degradation of cyanidin-3-O-glucoside and peonidin-3-O-glucoside in the presence of digestive enzymes [4]. Anthocyanins were unstable and degraded into phenolic acids in the simulated intestinal fluid [28].

In addition, during in vitro simulated digestion, the contents of quercetin and kaempferol increased due to the breakdown of flavonoid glycosides and the release of bound phenols [14]. The highest content of quercetin after digestion was in the Lr group (35.26 mg/L). However, anthocyanins might undergo ring fission and be degraded into phenolic acids during the in vitro simulated digestion process, decreasing the contents of peonidin-3-O-glucoside and cyanidin-3-O-glucoside [29].

However, after digestion, the content of phenols in the fermented groups of Lr and St was higher than that in the unfermented group. This indicated that LAB fermentation can increase the amount of phenolic compounds reaching the colon and improve the functional activity of phenolic compounds [25].

### 3.4. Effects of In Vitro Simulated Digestion on Antioxidant Activities

**Figure 2:**
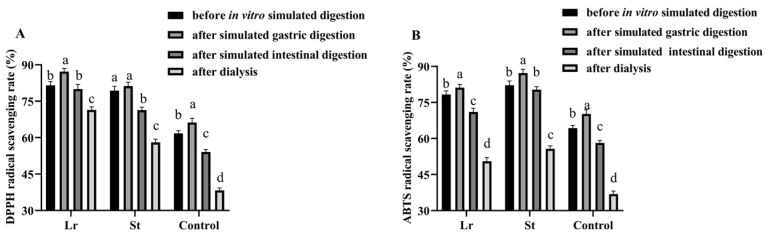
Changes in antioxidant activities of PSPJ during in vitro simulated digestion process. (**A**) DPPH and (**B**) ABTS. The values with different superscript letters in the same pattern column are significantly different (*p* < 0.05).

It has been shown that LAB fermentation promoted the release of phenols during the in vitro simulated digestion process, thereby increasing the antioxidant activities of the juice [30]. The DPPH radical scavenging rate increased from 61.80% to 81.53% and 79.35% after Lr and St fermentation, respectively (Figure 2). The ABTS radical scavenging rate, on the other hand, increased from 64.32% to 78.25% and 82.15% after Lr and St fermentation, respectively. The results showed that the antioxidant capacity of PSPJ can be improved by LAB fermentation.

The DPPH radical scavenging rate in the Lr and St groups increased from 81.53% and 79.35% to 87.19% and 81.21% after SGD and decreased from 87.19% and 81.21% to 80.02% and 71.31% after SID, respectively, during in vitro simulated digestion. In addition, the ABTS radical scavenging rate showed a similar trend. During the SGD, some bound polyphenols were released, thereby improving the antioxidant capacities [24]. However, during the SID, the decrease in antioxidant activities might be due to the increase in pH and degradation of phenols by digestive enzymes [31].

After in vitro simulated digestion, the fermented PSPJ had a stronger antioxidant capacity than the control group. TFC and TPC were positively correlated with antioxidant activity (Figure 2). The increase in antioxidant activities was attributed to the rise in the TPC and TFC.

However, this model of in vitro simulated digestion also had certain limitations, such as, firstly, difficulty in simulating the in vivo environment. Although the in vitro degradation test attempted to simulate the in vivo environment, it was still very different from the actual biological in vivo environment and might not fully reflect the degradation of materials in vivo. Secondly, the in vitro degradation test could not completely simulate the digestion and absorption process of animals and humans, so it was impossible to evaluate the actual degradation and absorption of materials in the body. Therefore, in order to accurately understand the biological activity of PSPJ in vivo, animal or human experiments needed to be further studied.

### 3.5. Principal Component Analysis (PCA) of Antioxidant Activities and Phenolic Profiles During In Vitro Simulated Digestion Process

**Figure 3:**
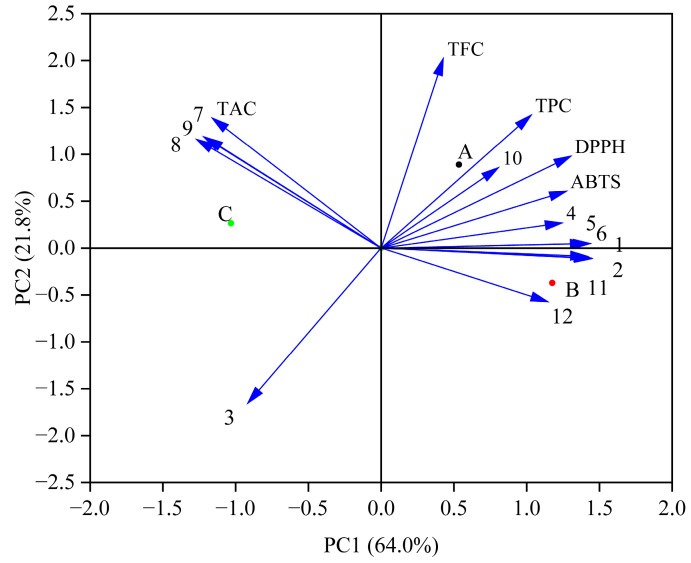
PCA of the antioxidant activities and phenolic profiles during in vitro simulated digestion. (A—the Group fermented by Lr, B—the Group fermented by St, C—the Control group; 1—Gallic acid, 2—Vanillic acid, 3—Syringic acid, 4—Protocatechuic acid, 5—Ferulic acid, 6—Caffeic acid, 7—Chlorogenic acid, 8—Peonidin-3-O-glucoside, 9—Cyanindin-3-O-glucoside, 10—Rutin, 11—Quercetin, 12—Kaempferol).

PCA indicated a total variance of 85.8% (64.0% for PC1 and 21.8% for PC2) (Figure 3). The changes in phenolic compounds may affect the antioxidant activity. Previous studies have shown that phenolic acids (caffeic acid and gallic acid) in blueberry juice fermented by Lactobacillus plantarum have high antioxidant activity [19]. The results showed that the contents of protocatechuic acid, ferulic acid, caffeic acid, and rutin directly affected the DPPH and ABTS free radical scavenging activities. Increased levels of these substances can affect antioxidant activity. Moreover, TPC and TFC were positively correlated with DPPH and ABTS free radical scavenging activities, as shown in the previous analysis (Table 2).

### 3.6. Changes in α-Glucosidase Inhibition Rate During Fermentation

**Figure 4:**
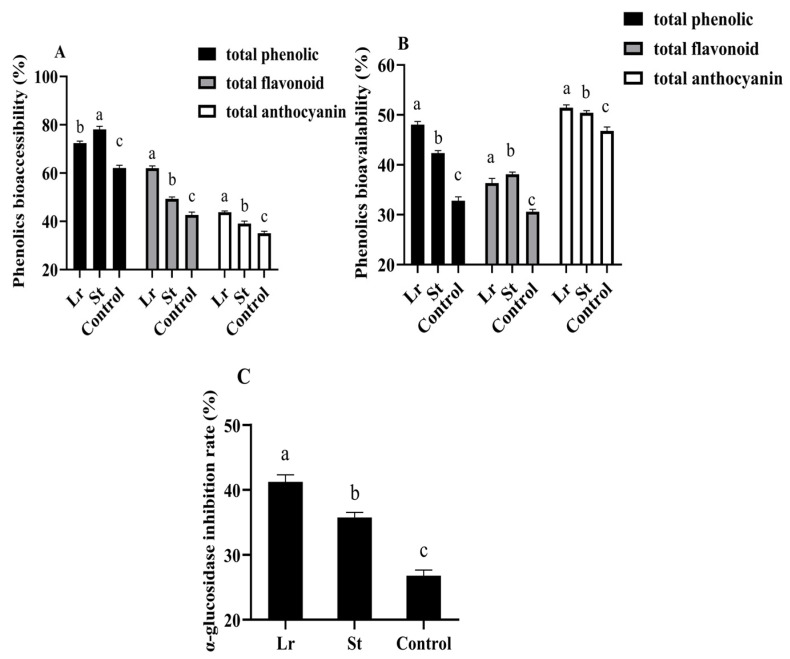
Changes in phenols bioaccessibility (**A**), phenols bioavailability (**B**), and α-glucosidase inhibition rate (**C**) of PSPJ. Different superscript letters come from values in the same pattern column that are significantly different (*p* < 0.05).

Studies have shown that the inhibition of α-glucosidase activity could slow the breakdown of carbohydrates to inhibit postprandial blood glucose rise [15]. Lr and St fermentation greatly improved the α-glucosidase inhibition rate from 26.78% to 41.21% and 35.75% (Figure 4), respectively. Phenolic acids inhibited the activities of several digestive enzymes, including α-glucosidase, as previously reported in the literature [32]. Therefore, after fermentation, the increase in phenolic acids content could increase the α-glucosidase inhibition rate and delay carbohydrate digestion, thereby achieving a hypoglycemic efficacy. As a result, Lactobacillus fermentation could enhance the hypoglycemic efficacy of PSPJ.

### 3.7. Changes in Phenols Bioaccessibility and Phenols Bioavailability During In Vitro Simulated Digestion Process

In this study, phenols bioaccessibility and phenols bioavailability in PSPJ were evaluated by measuring the changes in TPC, TFC, and TAC during the in vitro simulated digestion process. As shown in Figure 4, the total phenolic bioaccessibility increased from 62.09% to 72.42% and 78.11% in the Lr group and St group, respectively. The bioaccessibility of total flavonoid and total anthocyanin also increased. The decrease in pH during fermentation enhanced the stability of phenols, thereby increasing their bioaccessibility [33,34]. However, the total phenolic bioavailability increased by 15.22% and 9.54% in the Lr and St groups, respectively. It was previously reported that LAB fermentation could degrade phenolic substances of blueberry puree into more easily absorbed forms, thereby improving their bioavailability [4].

## 4. Conclusions

In this study, LAB fermentation increased the TPC, TFC, and α-glucosidase inhibition activity. The improvement of the antioxidant activity of PSPJ was due to the change in the phenolic composition of PSPJ by LAB fermentation. The improvement of phenolics’ bioaccessibility and bioavailability in PSPJ is attributed to the biotransformation of phenolics during fermentation. Furthermore, after simulated in vitro digestion, the viable bacterial counts from Lr and St exceeded 7 log CFU/mL. In summary, LAB fermentation could make PSPJ have better biological activity, such as phenols bioaccessibility, phenols bioavailability, and antioxidant activity of PSPJ. Notably, the biotransformation mechanism of phenolic substances in the in vitro simulated digestion process is not yet clear and still needs further study.

## Figures and Tables

**Table 1:** Phenolic profiles in PSPJ during in vitro simulated digestion process.

	Control			Lr			St		
	Before Digestion	After SGD	After SGD + SID	Before Digestion	After SGD	After SGD + SID	Before Digestion	After SGD	After SGD + SID
Gallic acid	4.11 ± 0.12 b	2.30 ± 0.29 c	8.11 ± 0.31 a	14.05 ± 0.11 b	11.21 ± 0.11 c	18.61 ± 0.12 a	8.75 ± 0.15 c	10.98 ± 0.14 b	15.61 ± 0.12 a
Vanillic acid	ND	ND	3.15 ± 0.07 a	7.42 ± 0.06 b	3.21 ± 0.09 c	8.61 ± 0.07 a	13.02 ± 0.08 b	6.13 ± 0.12 c	15.13 ± 0.09 a
Syringic acid	43.21 ± 0.09 c	47.21 ± 0.05 b	52.46 ± 0.21 a	26.13 ± 0.09 b	22.25 ± 0.17 c	33.12 ± 0.06 a	31.28 ± 0.17 c	37.36 ± 0.13 b	41.22 ± 0.16 a
Protocatechuic acid	0.48 ± 0.03 a	0.23 ± 0.02 b	ND	3.13 ± 0.05 c	6.23 ± 0.04 b	8.01 ± 0.12 a	5.08 ± 0.13 b	7.35 ± 0.11 a	3.05 ± 0.06 c
Ferulic acid	6.58 ± 0.06 c	11.32 ± 0.06 a	7.84 ± 0.14 b	17.23 ± 0.03 c	23.21 ± 0.13 a	21.23 ± 0.26 b	11.25 ± 0.03 c	16.11 ± 0.23 b	19.17 ± 0.13 a
Caffeic acid	4.67 ± 0.05 c	6.21 ± 0.07 b	11.45 ± 0.19 a	24.24 ± 0.18 c	27.11 ± 0.09 b	37.14 ± 0.17 a	15.37 ± 0.17 b	13.21 ± 0.16 c	27.15 ± 0.08 a
Chlorogenic acid	72.23 ± 0.19 a	69.25 ± 0.05 b	57.14 ± 0.16 c	66.19 ± 0.05 a	56.25 ± 0.23 c	58.12 ± 0.14 b	62.38 ± 0.16 a	54.12 ± 0.15 b	51.63 ± 0.07 c
Peonidin-3-O-glucoside	6.79 ± 0.02 a	5.21 ± 0.07 b	1.67 ± 0.15 c	4.59 ± 0.08 a	4.12 ± 0.05 b	0.67 ± 0.08 c	3.03 ± 0.04 a	2.04 ± 0.03 b	ND
Cyanidin-3-O-glucoside	2.15 ± 0.07 a	1.78 ± 0.11 b	ND	1.46 ± 0.04 a	0.23 ± 0.02 b	ND	1.12 ± 0.05 b	0.11 ± 0.02 c	ND
Rutin	13.37 ± 0.24 b	18.13 ± 0.10 a	8.97 ± 0.21 c	16.57 ± 0.18 b	21.32 ± 0.27 a	12.57 ± 0.32 c	15.13 ± 0.24 b	8.82 ± 0.14 c	20.23 ± 0.18 a
Quercetin	15.11 ± 0.13 b	11.21 ± 0.12 c	18.32 ± 0.28 a	28.45 ± 0.23 b	12.63 ± 0.19 c	35.26 ± 0.07 a	22.79 ± 0.18 b	15.36 ± 0.20 c	29.15 ± 0.12 a
Kaempferol	3.05 ± 0.03 c	7.21 ± 0.15 a	4.21 ± 0.07 b	5.16 ± 0.13 c	9.21 ± 0.06 a	6.53 ± 0.16 b	7.03 ± 0.13 c	8.01 ± 0.06 b	15.12 ± 0.08 a

“ND”: not detected. Values in the same row with different superscript letters are significantly different (*p* < 0.05).

**Table 2:** Person’s correlation coefficients of phytochemical concentration and antioxidant activities.

	TPC	TFC	TAC	DPPH	ABTS
TPC	1				
TFC	0.725 *	1			
TAC	0.335	0.536	1		
DPPH	0.826 **	0.607 *	0.096	1	
ABTS	0.936 **	0.502 *	0.064	0.726 *	1

* *p* < 0.05, ** *p* < 0.01.

## Data Availability

The original contributions presented in the study are included in the article; further inquiries can be directed to the corresponding author.
